# UV-Femtosecond-Laser Structuring of Cyclic Olefin Copolymer

**DOI:** 10.3390/polym14142962

**Published:** 2022-07-21

**Authors:** Kay Bischoff, Dominik Mücke, Gian-Luca Roth, Cemal Esen, Ralf Hellmann

**Affiliations:** 1 Applied Laser and Photonics Group, University of Applied Sciences Aschaffenburg, Würzburger Straße 45, 63743 Aschaffenburg, Germany; s160650@th-ab.de (D.M.); gian-luca.roth@th-ab.de (G.-L.R.); ralf.hellmann@th-ab.de (R.H.); 2 Applied Laser Technologies, Ruhr-University Bochum, Universitätsstraße 150, 44801 Bochum, Germany; esen@lat.rub.de

**Keywords:** femtosecond pulse laser, ultraviolet laser, cyclic olefin copolymer, laser ablation, microfluidics

## Abstract

We report on the laser ablation of cyclic olefin copolymer using an amplified ultrashort pulsed laser in the ultraviolet spectral range. In addition to a high ablation depth per laser-structured layer up to 74 μm at a fluence of 22 J cm${}^{-2}$, an excellent mean roughness $R_{a}$ of laser-patterned surfaces down to 0.5 μm is demonstrated. Furthermore, with increasing fluence, increasing ablation efficiencies up to 2.5 mm^3^ W^−1^ min^−1^ are determined. Regarding the quality of the ablation, we observed steep ablation flanks and low debris formation, though for fluences above 10.5 J cm${}^{-2}$ the formation of troughs was observed, being attributed to multiple reflections on the ablation flanks. For comparison, laser ablation was performed under identical conditions with an infrared laser wavelength. The results highlight that UV ablation exhibits significant advantages in terms of ablation efficiency, surface roughness and quality. Moreover, our results show that a larger UV focus spot accelerates the ablation process with comparable quality, paving the way for high-power UV ultrashort pulsed lasers towards an efficient and qualitative tool for the laser machining of cyclic olefin copolymer. The production of complex microfluidics further underlines the suitability of this type of laser.

## 1. Introduction

Since its introduction in the 1990s, the technical polymer cyclic olefin copolymer (COC) has enabled numerous applications in, e.g., photonics, sensing and medical technologies [1]. In addition to a high optical transparency and a low water absorption, this material exhibits a high glass transition temperature, chemical resistance and proven bio-compatibility [2,3,4,5,6]. With these properties, it is superior to standard polymers such as polymethylmethacrylate (PMMA) or polycarbonate (PC), while also being compatible with economic manufacturing and processing methods such as injection molding, hot stamping [7,8] and micromilling [3,9]. Several laser-based manufacturing processes have been introduced to produce internal [10] and external [11,12,13] microfluidic channels and integrated optical elements such as waveguides or Bragg gratings [14,15], to electrify COC-based lab-on-chip systems [16], or to weld transparent substrates without additional absorbing layers [17]. COC-based systems for contactless dielectrophoresis [18], protein separation [19], H${}_{2}$ sensing [20], or a dye laser [21] have been, amongst others, implemented so far.

Laser ablation of transparent polymers such as PMMA [22,23,24,25], PHEMA [26], PEEK [27] or PE [28] has already been extensively studied. For COC, however, there are only few reports, mainly based on using CO${}_{2}$ [11,12], excimer [29] or infrared (IR) solid-state lasers in the ns [13] or fs pulse regime [30,31]. Especially for polymers, the usage of ultrashort laser pulses in the femtosecond range exhibits many advantages in micromachining by offering low ablation thresholds, low thermal effects and heat-affected zones, as well as low debris formation [32], respectively.

Besides the pulse duration, the wavelength of the laser is one of the essential factors for laser material processing. Based on the development of high-power ultra-short pulsed laser systems, and the increased lifetime and the availability of third harmonic generation (THG) modules with high conversion efficiency and beam quality, systems are nowadays available that fulfill the requirements of laser micromaterial processing in industrial applications.

Generally, using a shorter laser wavelength introduces several advantages to the process. First of all, the propagation of the laser beam in the focal area is affected by its wavelength. Smaller focal diameters $d_{f}$, calculated by:

(1) $$d_{f}=2\omega_{0}=\frac{4\lambda }{\pi }\;\cdot \;\frac{f}{D}\;\cdot \;M^{2}$$

with the beam waist $\omega_{0}$, raw beam waist *D*, laser wavelength λ, focal distance *f* and beam parameter product $M^{2}$ can be achieved. In addition, the Rayleigh length $Z_{r}$ of the beam calculated by:

(2) $$Z_{r}=\frac{\pi \;\cdot \;\omega_{0}^{2}}{\lambda \;\cdot \;M^{2}}$$

is reversely proportional to the wavelength, which is associated with a higher process stability [33]. Secondly, a shorter wavelength is equivalent to a higher photon energy, which can break the chemical bonds of the irradiated material more easily, especially in materials with a large band gaps, such as, e.g., transparent polymers. Both advantages enable a lowering of the ablation threshold, an improved ablation quality and efficiency and have already been demonstrated in different applications for various materials, other than polymers [22,24,26,28].

In this study, the advantages of ultraviolet (UV) ultrashort-pulse (USP) lasers over near-infrared (NIR) lasers in processing transparent COC are reported. Besides the combination of ultrashort pulses and a UV wavelength, we highlight the usage of a regenerative pulse-amplification laser system with significantly higher repetition rates and accompanying processing speeds as compared to common Ti:Sa lasers. The laser process is studied by measuring ablation depths, ablation efficiencies, resulting surface roughness and qualities by employing different analysis methods. Moreover, obtained results are directly compared to IR laser micromachining.

## 2. Materials and Methods

### 2.1. Design and Characterization of the Laser Machine

**Figure 1:**
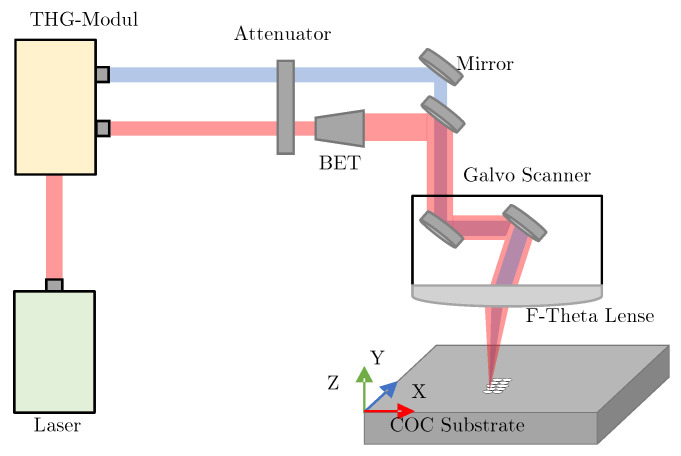
Schematic illustration of the laser-machining setup with the wavelengths 343 nm and 1030 nm.

For the experiments, an ultrashort pulsed laser (Pharos 10-600-PP, Light Conversion) was used. The laser emits at a fundamental wavelength of 1030 nm (IR) with a minimum pulse duration of 220 fs and an adjustable repetition rate of up to 610 kHz. An integrated third harmonic module can output a wavelength of 343 nm (UV). Figure 1 illustrates the structure of the laser-machining setup and Table 1 summarizes its specification for the IR and UV wavelength. The laser power is varied by an attenuator. A galvanometric scanner unit (Rothor AR800, Newson Engineering) deflects the laser to the desired location and a telecentric f-Theta lens with a focal length of 100 mm (Qioptiq Linos) focuses it.

**Figure 2:**
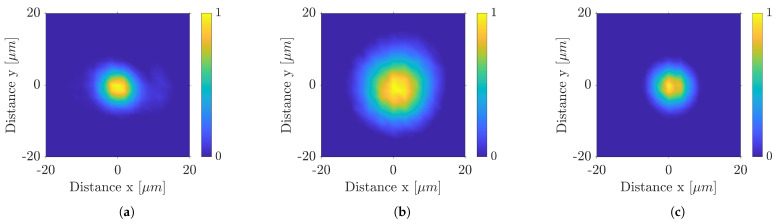
Focal points of the wavelength in (**a**) UV Z = 0 mm (**b**) UV defocused (Z = −1 mm) (**c**) IR with BET (Z = 0 mm).

The laser power measurement was performed using a power sensor at sample level. The size of the focal point, power distribution and Rayleigh length were determined experimentally using a monochrome camera (UI-1490S, IDS—pixel size: 1.67 μm). At UV wavelength, the focus spot size measures approx. 15 μm (cf. Figure 2a). Different focal spot sizes can only be compared with each other to a limited extent; otherwise, the differences in the results cannot be attributed only to the wavelength. Thus, a beam expander telescope (BET) was installed for the IR setup, expanding its raw beam diameter to reduce the focal spot size to the same as in UV (cf. Figure 2c). The Rayleigh length $z_{R}$ of IR wavelength (with BET) is about 180 μm and of the UV laser is about 500 μm. Defocusing by 1 mm increases the focus diameter in the UV to 30 μm (cf. Figure 2b).

### 2.2. Implementation and Evaluation of the Ablation Studies

For the experiments, COC plates (TOPAS 6017S) measuring 20 mm× 20 mm× 1.5 mm were used. The glass-transition temperature of the polymer is 175 ${}^{\circ }C$, surface roughness is 26 nm and a band gap of approximately 4 eV was measured by Tauc method [34].

**Figure 3:**
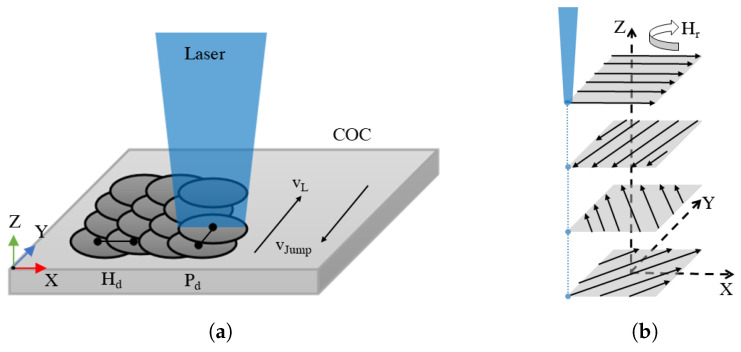
(**a**) Schematic representation of laser ablation with pulse spacing $P_{d}$, hatch spacing $H_{d}$, scan velocity $v_{L}$, jump velocity $v_{Jump}$. (**b**) Representation of the hatch rotation and dynamic refocusing.

An area of 1 mm${}^{2}$ was ablated using 10 passes, each rotated by 100 ${}^{\circ }$ (no double passes) with respect to the previous pass. Hatch rotation ($H_{r}$) (cf. Figure 3) was applied to obtain a uniform ablation with no preferred direction. Due to this small ablation area, it must be considered that thermal accumulation can distort the ablation results. On the one hand, heated material is usually easy to ablate, leading to increased efficiencies. On the other hand, negative effects can also occur due to high temperatures, resulting in defects. By comparing the jump velocities $v_{Jump}$ (cf. Figure 3a, 100 mm s^−1^ and 1000 mm s^−1^), the influence of this parameter on the ablation quality was determined within preliminary studies. The resulting surface showed large area fusion at COC with a larger $v_{Jump}$, since the time between two line scans was smaller. Therefore, to allow sufficient cooling and minimize the factor of thermal accumulation, a unidirectional ablation strategy with a jump speed of 100 mm s^−1^ was chosen.

**Figure 4:**
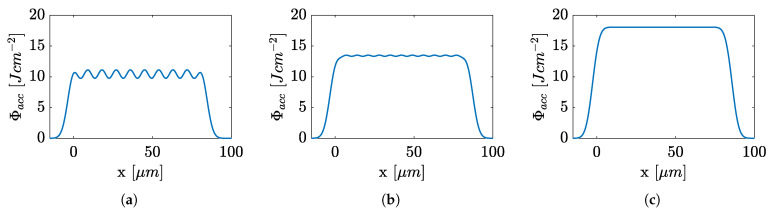
Simulated 2D cumulative fluence in pulse or hatch directions with overlap of (**a**) 35% (**b**) 50% (**c**) 65% for a single pulse fluence of 10 J cm^−2^ following [35].

Depending on the pulse overlap (percent of diameter), different cumulative energy inputs into the material were obtained. By a pulse overlap of > 50% the intensity peaks of the single Gaussian spots were flattened. Figure 4 shows the simulated cumulative fluence at the percentage pulse overlaps smaller and larger than 50%. Uniform energy input is necessary for good roughness. For this reason, a pulse overlap of 65% was chosen, as shown by Schwarz et al. [35]. This pulse overlap combines good resulting surfaces due to a homogeneous energy input with a good process time. This overlap was performed in both scan and hatch directions. The distance between the individual hatch lines was, thus, equal to the pulse spacing. The surface was roughened before ablation due to the transparency of the specimen; otherwise, the optical properties would change during ablation process. This process lowers the ablation threshold. A jump speed of 1000 mm s^−1^ and 50% pulse and 50% hatch overlap of the following ablations were chosen as parameters for the roughening. Through a preliminary study, the necessary fluence was determined to be 0.5 J cm^−2^ for a resulting roughness of 0.7 μm.

In addition, the reduction in fluence with increasing ablation depth caused by defocusing was counteracted by refocusing per pass by moving the Z axis. The degree of refocusing was determined on the basis of a preliminary study without refocusing or by a single removal.

For the experiments, the laser power was varied for the individual ablations. After the process, the samples were placed in an ultrasonic bath for approximately 10 min to remove loose particles and contaminants. Optical inspection of the specimens was performed with the digital microscope (Leica DMV6). High-resolution images were taken by a scanning electron microscope (MAIA3, Tescan). For this purpose, the samples were coated with a conductive layer by sputtering. The evaluation of the depths and roughness of the ablations was performed using a laser scanning microscope (VK-X200, Keyence). The depth of ablation was determined in areas of 1416 μm × 1062 μm and the average surface roughness $R_{a}$ was validated along lines in areas of 250 μm × 200 μm. Due to a deep ablation, samples had to be abraded after the ablation depth measurement in order to measure the resulting surface roughness. Using the ablation depths and process parameters, the ablation of a single pulse $V_{sp}$ was determined computationally [36]:

(3) $$V_{sp}=\frac{d\;\cdot \;H_{d}\;\cdot \;P_{d}}{N_{l}}$$

where *d* is the ablation depth, $H_{d}$ the hatch distance or $P_{d}$ the pulse distance and $N_{l}$ the number of passes of an ablation. Combining this with the repetition rate of the laser $f_{L}$ gives the ablation rate of the process:

(4) $$MRR=V_{sp}\;\cdot \;f_{L}$$

The material removal rate (MRR) was used to evaluate the ablation by defocused UV laser spot in Section 3.3 [36]. With additional consideration of the power, we considered the ablation efficiency (AE) in mm^3^ W^−1^ min^−1^ with the relationship:

(5) $$AE=\frac{MRR}{P_{av}}=\frac{d\;\cdot \;H_{d}\;\cdot \;P_{d}}{N_{l}}\;\cdot \;\frac{f_{L}}{P_{av}}$$

where $P_{av}$ is the power of the laser beam on the sample [37].

In a first study, the ablation behavior of COC was investigated during processing with the UV laser. Subsequently, a comparative study was carried out under the same conditions with the IR wavelength. The roughening with 1.4 J cm^−2^ produced a surface roughness of about 1 μm. Finally, the material was ablated with a defocused UV spot (approx. 30 μm, cf. Figure 2b). To obtain the desired focal diameter, the focus was moved 1.45 mm towards the sample. All experiments were performed under the same conditions and with 65% pulse and hatch overlap. By the applied defocusing, a fluence of up to 6 J cm^−2^ can be realized.

## 3. Results and Discussion

### 3.1. Ablation by Focussed UV Laser

**Figure 5:**
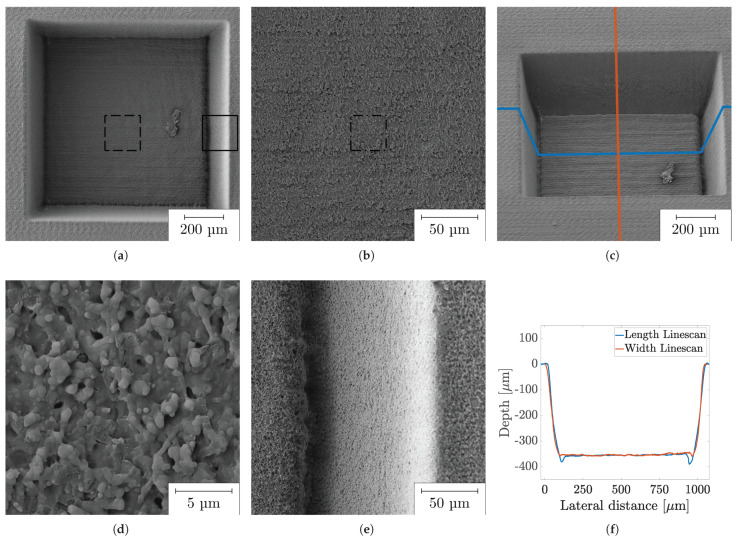
SEM images of an ablation cavity on COC with a fluence of 10.5 J cm^−2^ in different magnifications: (**a**) overview; (**b**) magnified section of (**a**) (dashed line); (**c**) tilted image; (**d**) magnified section of (**b**); (**e**) magnified section of (**a**) (solid line) with trough; (**f**) line scans in X and Y direction of (**c**).

First, COC surfaces were ablated by the focussed UV laser. Figure 5 shows the ablated topography in various magnifications using a fluence of 10.5 J cm^−2^. When processing COC with the UV laser, clearly defined edges can be created (cf. (a)). The results shown clearly reveal that the UV-USP laser is a suitable tool for processing COC. As an ablation mechanism, degradation as a combination of oxidation and dehydrogenation has been discussed by Suriano et al. [31] and Yang et al. [38]. The relationship between ablation and fluence has been reported for COC by Leech et al. [39] and Maccann et al. [13].

**Figure 6:**
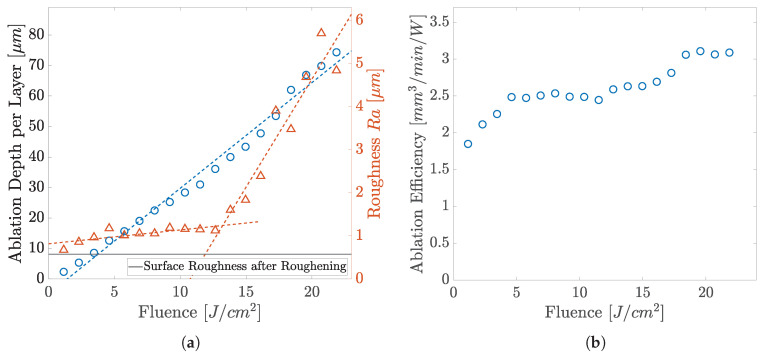
Ablation values of COC by the UV laser: (**a**) ablation per pass and roughness; (**b**) ablation efficiency.

**Figure 7:**
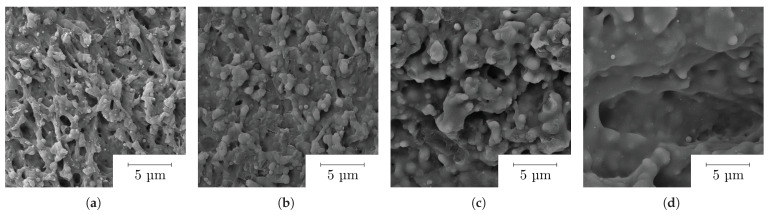
SEM image of the topography evolution of COC by the focused UV laser at the fluence of (**a**) 4.6 (**b**) 10.5 (**c**) 16.8 (**d**) 22 J cm^−2^.

The relationship of the ablation depth per pass and the roughness of the resulting surface after ablation with increasing fluence is shown in Figure 6a. Graph (b) depicts the ablation efficiency, revealing a continuous increase with fluence. The roughness increase can be well interpreted on the basis of the topography development shown in Figure 7. The SEM studies indicate that thermalisation shapes the surface topography due to increasing overall heat input at higher fluences and heat accumulation. A simple way to show the occurrence of heat accumulation is to calculate the critical frequency (pulse repetition rate) [40,41]:

(6) $$f_{cr}=\frac{D_{th}}{d_{spot}^{2}}$$

with the thermal diffusivity of COC $D_{th}$ ( $9\times 10^{-8}$ $m^{2}$/$s^{-1}$ [2]) and the diameter of the laser spot (14.5 μm). In the case of our experiments, we obtained a critical frequency of 428 Hz which indicates that heat accumulation must have a large influence at the frequency of 50 kHz, although there is only a small amount of heat input when ablating with ultrashort laser pulses.

At low fluences, cold ablation takes place, which is typical for ultrashort laser pulses. With increasing fluence, more material is ablated and a rugged surface structure results from the abrupt solidification of the material softened or molten during laser irradiation. However, the polymer melt does not persist long enough to even the surface or to close small holes. At the previously lowest fluences, emerging sharp structures are becoming more and more rounded at moderate fluences and previously visible voids are filled by molten material, resulting in a low surface roughness. This behavior is evident up to a fluence of 14 J cm^−2^ and the quality deteriorates as the fluence increases. The value for $R_{a}$ rises linearly with low gradient for low fluences up to 14 J cm^−2^ from 0.6 μm up to 1.1 μm. If the fluence is further increased, the thermal effects are more intensified, while the material removal also increases. The maximum ablation per layer of 70 μm is achieved by using a fluence of about 22 J cm^−2^. The quality of the resulting surface decreases due to fusion caused by the high energy input. The sub-micrometer structures visible at low fluences are almost completely rounded by the melt and significantly coarser structures are created, which is also represented in the strong linear increase in the surface roughness with a higher gradient.

These findings highlight the advantage of using ultrashort laser pulses in material processing, as it is shown that the accumulation of heat negatively affects the ablation quality and it is considered that the effect of thermalisation and the increase in roughness with longer pulses already occurs at lower fluences. Heat accumulation arises from both interpulse and intrapulse effects. The accumulation of heat can be counteracted, for example, by lowering the overall processing time with a lower repetition rate. Low repetition rates are applied in the literature when machining COC and polymers in general [12,13,29,30,31]. If a larger area is machined, the influence of intrapulse heat accumulation on the removal rate is lowered, since the material has a longer cooling phase between ablation passes.

With increasing fluence, unwanted ablation geometries emerging in the form of ridges on the edges and a trough on the lower flank of the ablation (cf. Figure 5e) appear. These troughs are also illustrated in linescans of the LSM images (cf. (f)), their formation appearing at fluences of 4 J cm^−2^ to about 18 J cm^−2^. Depending on the fluence applied, the depth of the trough is up to 13% of the total ablation depth. Small fluences cause smaller troughs. It is worthwhile to note that if the hatch direction of the first layer is rotated by 90 ${}^{\circ }$, the trough is formed on the lower flank instead of the right side. One approach to explain the trough on the flank is the interaction of the partially reflected beam, which has sufficient power to produce a renewed ablation and a multiple reflection. This behavior has also been observed on other materials [42]. The intensity of the reflected beam can be calculated by Fresnel’s formulas [43]. This behavior occurs due to increased ablation caused by a generated heat front after the first pass. After the first pass, the material is already heated up, which means that there is no longer such a high fluctuation in temperature, producing a more homogeneous material removal. The occurrence on several flanks can be explained conditionally by the rotation of the hatch. This behavior occurs at fluences from 4 J cm^−2^ to about 18 J cm^−2^. The omission of the phenomenon above a fluence of 18 J cm^−2^ can be attributed to an increasing degree of fusion and the resulting increase in the flank angle.

The ablations do not exhibit any bulges adjacent to the ablations as observed in COC processing using a CO${}_{2}$ laser [11]. Similarly, there are no bulges, bubble-like formations, or mergers, as reported using NIR nanosecond lasers [13], although the repetition rate and scan speeds are significantly higher in our experiments. Even though a much smaller repetition rate is used in experiments by Wang et al. [30] with a Ti:Sa laser ( 800 nm, 120 fs, 1 kHz), at the lower fluence range the measured roughness (1 μm to 2.5 μm) is in a similar range to our experiments, but no increase to values above 5 μm is recorded in this study at high fluences. Although the experiments of Suriano et al. [31] using shorter laser pulses ( 800 nm, 40 fs, 1 kHz) and a lower repetition rate showed lower resulting roughnesses (<0.2 μm), the material removal rates shown by us exceed the values shown there (approx. 0.3 mm^3^ W^−1^ min^−1^) by a multiple.

### 3.2. Comparison of Infrared and Ultraviolet Laser Ablation

**Figure 8:**
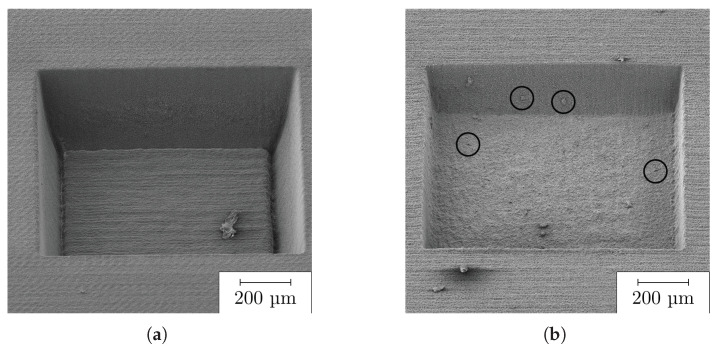
A 45${}^{\circ }$-tilted SEM image of ablation cavities on COC with different wavelengths: (**a**) UV; (**b**) IR under same conditions at a fluence of 10.5 J cm^−2^ (circles show reattached particles).

In the further course of the study, we compared the behavior of COC when processed by ultraviolet and infrared ultrashort pulsed laser radiation. Figure 8 compares the ablated topographies with (a) the UV laser and (b) the IR laser for identical applied fluences of 10.5 J cm^−2^. Apparently, ablation with the IR laser shows, similar to the UV, formation of ridges and troughs on the flanks and melting of the COC at higher fluences, too. The trough formation starts at a fluence of 10.5 J cm^−2^. As the fluence increases, these troughs deepen in absolute values but also proportionate to the ablation depth. If similar depths of ablation are compared, the maximum depths of the troughs in the UV are slightly smaller than in the IR. The need for lower fluences for the evolution of troughs at UV can be explained by a higher percentage of the reflected beam. Due to dispersion, COC has an increasing refractive index with decreasing wavelength [2]. According to Fresnel’s formulas, the reflected fraction is larger due to impingement on a material with larger refractive index [43]. In addition, we find larger and more reattachments of particles (debris) when ablation is performed by infrared laser radiation (cf. Figure 8). As the fluence increases, a larger burr forms on the edges, regardless of the ablating laser wavelength.

**Figure 9:**
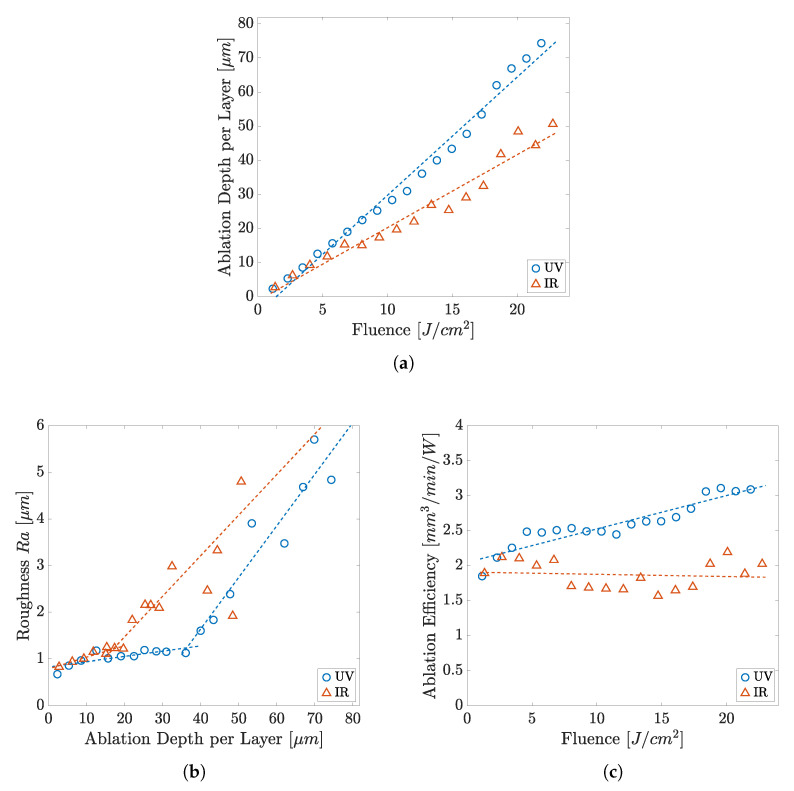
Comparison of the ablation values of COC of the different wavelengths UV and IR under same conditions: (**a**) ablation (**b**) roughness (**c**) ablation efficiency.

The results of the ablation study with the UV laser and the IR laser, performed on COC in focus position, are compared in Figure 9. Similar to the results in the UV, which, for comparative reasons, are also shown, the ablation per pass (a) with the IR laser shows a proportional relationship of ablation per pass and fluence, though revealing a lower increase with fluence. Ablation starts at a fluence of 1.3 J cm^−2^ and the largest ablation per pass of 50.7 μm is achieved at a fluence of 22.4 J cm^−2^.

The graph in Figure 9b compares the achieved roughness for UV and IR against ablation depth per pass. Lowest roughness with the IR laser is observed at a fluence of 1.3 J cm^−2^ being 0.8 μm, while at UV 0.6 μm (75% of IR) is reached. The roughness rises linearly with increasing energy input and measures 2 μm at a fluence of about 16 J cm^−2^. While low roughness of about 1 μm can be reached with ablation depths per layer of up to 38 μm with the UV laser, the roughness of the IR laser rises abruptly (second linear rising domain) at a much smaller depth of about 20 μm (52% of UV).

With the IR wavelength, the ablation efficiencies shown in Figure 9c are in the range of 1.5 mm^3^ W^−1^ min^−1^ to 2 mm^3^ W^−1^ min^−1^. While the ablation efficiencies stagnate at this value, we find a continuously growing efficiency up to 3.2 mm^3^ W^−1^ min^−1^ (160% of IR) with the UV laser.

When ablation of both wavelengths is compared, the greater ablation per pass and the resulting greater ablation efficiency of the UV laser becomes apparent. It must be noted that the fluences of the UV laser are generated by a THG module which, due to its conversion efficiency, must be pumped at a much higher power in the IR. For this reason, a significantly higher fluence can be achieved with the first harmonic of the laser than in the third harmonic. Nevertheless, a higher fluence is always accompanied by a higher energy input into the material, which has a negative effect on the thermal load of the sample. The influence of shorter wavelengths on the ablation threshold, ablation rate and quality has already been shown and discussed for transparent polymers before [22,27,28,44]. This is due to the fact that the higher photon energy at shorter wavelengths can either already lead to direct polymer bond breaking (photochemical processes) or shift the absorption process from multiphoton to two-photon absorption. While, at the band gap of COC (4 eV) for the applied ultraviolet wavelengths, a two-photon absorption is possible; for the applied infrared wavelength, the absorption of six photons is required. Although the probability of multiphoton absorption is comparatively high at pulse durations in the femtosecond regime, photons that do not participate in the ionization process take part in photothermal processes [31,32,45].

In addition to the higher quality and efficiency of ablation with the UV laser, process control must also be discussed with regard to industrial processes and machining systems. The process with the IR laser is more complex due to the higher number of necessary refocusings. Ablation with a UV laser is more flexible and robust. The greater processing reach can vary the thickness of substrates over a wider range, ablate deeper, or allow for greater positioning tolerance. In addition, process generation is also simpler, as the positioning of the substrate has a greater tolerance. In addition, costs of an industrial machine increases rapidly with increasing poitioning accuracy. However, it must be noted that a THG module causes costs and has a limited lifetime.

### 3.3. Ablation by Defocussed UV Laser

**Figure 10:**
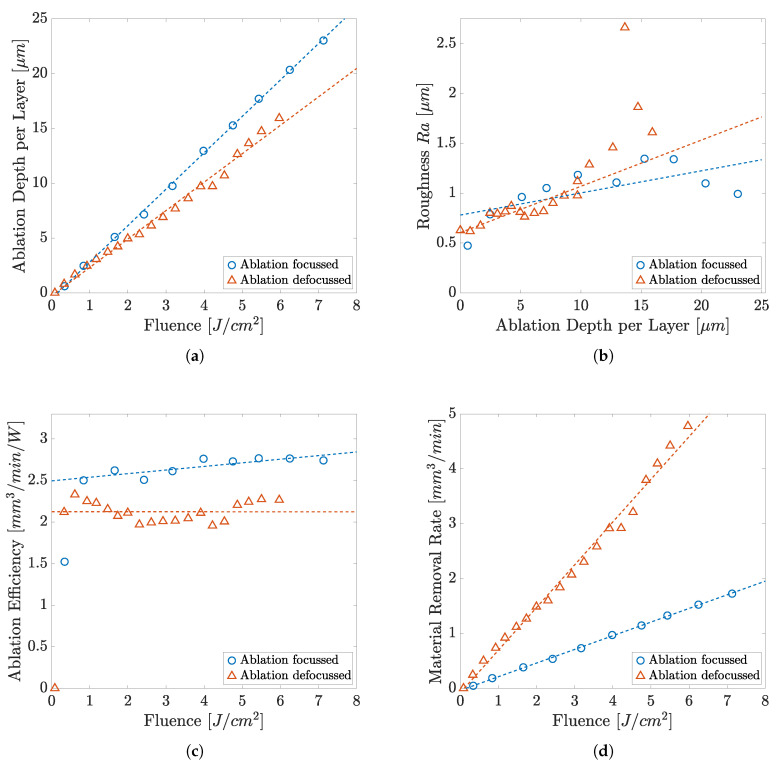
Comparison of the removal values of COC at UV with different focus positions: (**a**) material removal, (**b**) roughness, (**c**) removal efficiency, (**d**) removal rate plotted on fluence.

Due to the good ablation results using the UV laser, further studies with a defocused UV laser (spot size 30 μm) were performed. Figure 10a compares the removal depth per pass in relation to the fluence. Section (b) compares the roughness of the resulting surface. Graph (c) shows the efficiency of removal and (d) the depth of removal of the different fluences, respectively. Ablation occurs from 0.3 J cm^−2^ onwards with a proportional relationship between the fluence and the ablation per pass. Roughness $R_{a}$ of 0.6 μm to 2.6 μm can be achieved by this method. The maximum of the removal efficiency amounts to 2.33 mm^3^ W^−1^ min^−1^ at a near-threshold fluence of 0.6 J cm^−2^. The highest ablation per pass of 16 μm was attained at the maximum fluence. A trough forms on the flank of the ablation as the fluence increases.

Ablation of COC shows, by trend, a similar behavior for focused and defocused machining. However, the material removal per pass and the removal efficiency is about 20% higher under focused machining. For smaller fluences, defocused ablation shows a finer surface area with $R_{a}$ of about 0.77 μm. From a fluence of 4 J cm^−2^, the roughness of the resulting surface increases abruptly, whereas with focused ablation this increase only occurs at significantly higher fluences of about 10.5 J cm^−2^. The removal efficiency has its maximum at low fluences and saturates at 2.2 mm^3^ W^−1^ min^−1^ (79% of focused beam) as the fluence increases.

The factor relevant for process acceleration is the ablation rate shown in Figure 10d. The ablation rate of the defocused ablation is always higher than the ablation rate of the focused experimental series, once the threshold fluence is exceeded. Thus, with a fluence of 5.9 J cm^−2^, an ablation rate of 1.4 ${\mathrm{mm}}^{3}$min^−1^ is obtained with a focused ablation, whereas up to 6.9 ${\mathrm{mm}}^{3}$min^−1^ is realized by a defocused beam with a fluence of 6 J cm^−2^.

With increasing fluence, however, this acceleration of the ablation process only occurs with decreasing ablation efficiency. When comparing the development of the roughness values between defocused and focused ablation, it is noticeable that these show similar characteristic areas. In the case of defocused ablation, after an increase in roughness up to about 5 J cm^−2^, the graph also shows a slight dip to a lower roughness before it increases rapidly, but this effect is already observed at lower ablation rates as compared to the focused machining conditions. The same effect of thermalisation is assumed, in that rugged structures are rounded off by the prolonged presence of the melt before the roughness rises sharply due to excessive fusion. To explain this, the total power input into the material must be taken into account. Although comparable pulse fluences exist, resulting in similar ablations per pass, significantly more power is, overall, introduced into the material with the defocused laser spot. Accordingly, the heat can accumulate faster and the melting effects occur earlier. Coarser structures can be created by an accelerated process using the defocused beam, whereas precise patterning should be performed by a tighter focused beam. A potential application could be a sizing of the surface with a smaller fluence in the defocused state. These results open up the use of focusing optics with long focal lengths and accompanying large Rayleigh lengths for robust industrial processes with significantly lower requirements for horizontal workpiece positioning.

### 3.4. Exemple of a Microfluidic Structure

**Figure 11:**
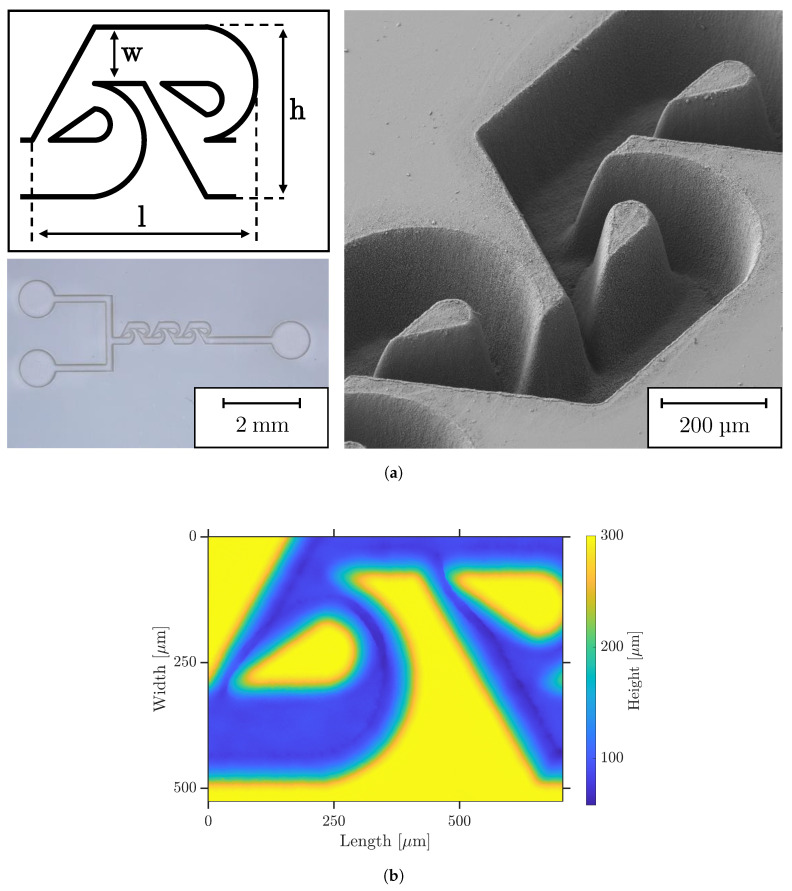
Exemplification of an UV USP laser ablated microfluidic Tesla mixer leaning on [48]. (**a**) Top left: design of one Tesla sturcture geometry with l = 790 μm, w = 200 μm and h = 600 μm. Bottom left: optical micrograph of one of eight mixers. Right: 45${}^{\circ }$-tilted SEM image. (**b**) LSM image.

To demonstrate the advantages of micromachining COC with the UV USP laser, we show a sophisticated application from microfluidics—the tesla mixer. It has been shown several times that tesla structures can efficiently mix two fluids in the size scale of microfluidics [46,47]. However, they exhibit a geometry and structure size at which a variety of fabrication methods reach their resolution limits. Following the results of Hossain et al. [48], we fabricate an array of eight 200 μm deep micromixers consisting of two inlet and one outlet (diameter 1 mm) channels (width 200 μm) and three tesla structures each, as shown in Figure 11a. Focused UV laser processing was further performed with rotation of the hatch direction ( 75 ${}^{\circ }$), 65% pulse and hatch overlap, and the fluence of 4.6 J cm^−2^. When the entire array is hatched, the jump speed can be increased to 1000 mm s^−1^ because there are larger jump paths when compared to the 1 × 1 mm^2^ test structures. For this reason, the temperature of the COC increases less, resulting in a reduction in the overall processing time. The desired depth was reached after 15 passes and no refocusing is required.

As shown by the optical microscopy and SEM in Figure 11a and LSM in (b), the structure can be fabricated with superior accuracy and shape fidelity. All the quality characteristics of the test structures from previous sections can be transferred to the produced microfluidics. Minimal discoloration of the polymer occurs solely at the edge of the fine structures within the Tesla mixer. There are no bulges or fusions and very little debris. The curves and tips of the tesla structure are very precisely pronounced due to the hatch rotation, although the contour of the geometry itself was not machined.

The exemplarily fabricated structure shows the high application potential of ultrashort pulsed lasers with ultraviolet wavelength for the shaping processing of COC such as the generation of microfluidics. On the one hand, the material undergoes a low heat deposition due to the ultrashort laser pulses in ultraviolet wavelength and high-quality ablations are generated. On the other hand, the structure can be fabricated with high resolution in a robust process due to the small focus spot at a large Reyleigh length. If even smaller structures with higher resolutions are required, the raw beam of the UV laser can be enlarged using a BET, so that the focus diameter can be drastically reduced.

## 4. Conclusions

In summary, this work demonstrates the ability of fast and high-quality micromachining COC by an amplified ultrashort pulsed laser in the ultraviolet wavelength range ( 343 nm). Ablation tests are investigated with respect to their process speed, efficiency, roughness and quality. Here, high ablation efficiency up to 3.2 mm^3^ W^−1^ min^−1^ was found with, at the same time, high edge quality and low debris adhesion and fusion. Micromachined structures exhibit a fine surface roughness of 1 μm to 2 μm over a large fluence range. Only at high fluences, however, does the roughness in ablated cavities increase rapidly due to fusion and trenching caused by heat accumulation. Nevertheless, these fluences can be used for future processes such as drilling or cutting.

The comparative ablation study with the laser in its fundamental wavelength under identical process conditions shows the superiority of the UV laser in terms of process speed, quality and efficiency due to the different thermalisation, absorption and ablation mechanisms at the IR and UV wavelengths. With the UV laser, 75% smaller surface roughness is measured in low fluence regime and a two-times deeper ablation per layer can be reached with same roughness. The UV laser increases ablation efficiency by up to 60%.

Motivated by the excellent ablation results with the UV, further tests were carried out with a doubled laser focus diameter. By distributing the laser power to a larger spot, higher scanning speeds and hatch distances can be used at lower fluences. On the one hand, the ablated structures show a similar high quality but about 20% smaller ablation efficiency as compared to focused process conditions. On the other hand, the processing speed can be increased by five times without deteriorating ablation quality, such as by the formation of troughs or excessive fusion.

To underline the significant advantages of UV USP laser micromachining, we exemplify the process using a complex microfluidic structure—the Tesla mixer. The previously found properties regarding quality of the resulting structures are fully transferred. Furthermore, the application shows how accurately such geometries can be transferred.

## Figures and Tables

**Table 1:** Specification of the laser system in the fundamental wavelength with BET and 3rd harmonic wavelength at optimized repetition rate.

Parameter	IR	UV	Unit
Wavelength	1030	343	n m
Power	2.4	2.1	W
Repetition rate	50		k Hz
Pulse width	220		f s
Beam quality (M${}^{2}$)	1.06	1.40	-
Raw beam diameter	10.5	4	m m
Focal length	100		m m
Focal diameter $1/e^{2}$X	14.6	14.1	μm
Focal diameter $1/e^{2}$Y	14.4	14.9	μm
Rayleigh length	180	500	μm

## Data Availability

Not applicable.

## Abbreviations

The following abbreviations are used in this manuscript:

BET	beam expander telescope
COC	cyclic olefin copolymer
IR	infrared
LSM	laser scanning microscope
SEM	scanning electron microscope
THG	third-harmonic generation
USP	ultra short pulsed
UV	ultraviolet
